# Discussions of Antibiotic Resistance on Social Media Platforms: Text Mining and Mixed Methods Content Analysis Study

**DOI:** 10.2196/37160

**Published:** 2025-04-25

**Authors:** Jocelyne Arquembourg, Philippe Glaser, France Roblot, Isabelle Metzler, Mélanie Gallant-Dewavrin, Hugues Feutze Nanguem, Adel Mebarki, Paméla Voillot, Stéphane Schück

**Affiliations:** 1 Laboratoire l3, Telecom Paris, Telecom Paris, Palaiseau, France; 2 Unité EERA Institut Pasteur, CNRS UMR3525, Université de Paris, Paris, France; 3 CHRU Poitiers, INSERM U1070, CHRU Poitiers, Poitiers, France; 4 Association France Spondyloarthrites, Association France Spondyloarthrites, Tulle, France; 5 Association HTaPFrance, Association HTaPFrance, Meursault, France; 6 CREDIMI: Center for Research on International Market and Investment Law, University of Burgundy, Dijon, France; 7 Pfizer, Paris, France; 8 Kap Code, 146 Rue Montmartre, Paris, 75002, France, 33 625530241

**Keywords:** antibiotic ineffectiveness, antibiotic resistance, health-related quality of life, real-world, social media, quality of life, quantitative, qualitative, app, application, online, medical information, French, users, antibiotic, social media use

## Abstract

**Background:**

With the increasing popularity of web 2.0 apps, social media has made it possible for individuals to post messages on antibiotic ineffectiveness. In such online conversations, patients discuss their quality of life (QoL). Social media have become key tools for finding and disseminating medical information.

**Objective:**

To identify the main themes of discussion, the difficulties encountered by patients with respect to antibiotic ineffectiveness and the impact on their QoL (physical, psychological, social, or financial).

**Methods:**

A noninterventional retrospective study was carried out by collecting social media posts in French language written by internet users mentioning their experience with antibiotics, and the impact of their ineffectiveness on their QoL. Messages posted between January 2014 and July 2020 were extracted from French-speaking publicly available online forums.

**Results:**

A total of 3773 messages were included in the analysis corpus after extraction and filtering. These messages were posted by 2335 individual web users, most of them being women around 35 years of age. Inefficacy of treatment options and the lack of information regarding the use of antibiotics were among the most discussed topics. QoL was discussed in 63% of the 3773 messages posted. The most common is the physical impact (78%). Patients discussed the persistence of symptoms and adverse effects. The second kind of impact is psychological (65%), characterized by feelings of anxiety or despair about the situation.

**Conclusions:**

This social media analysis allowed us to identify a strong impact of the perceived ineffectiveness of antibiotic therapy on patients’ daily life particularly in terms of physical and psychological consequences. These results provide health care experts information directly generated by patients regarding their own experiences. Social media studies constitute a complementary source of evidence that could be used to optimize messages to the public about appropriate use of antibiotics.

## Introduction

When antibiotics were first introduced in the middle of the 20th century, they were hailed as wonder drugs. Patients and physicians alike were amazed at the almost miraculous effect of these drugs on serious bacterial infections. For the past 70 years, physicians have come to expect that antibiotics would cure almost all their patient’s bacterial infections, and patients expect that the miracle drugs will still work wonders. Despite an increasing rate of antibiotic resistance globally, they remain one of the most cost-effective, life-saving medicines contributing to an extended lifespan [1].

Commonly prescribed antibiotics are becoming less effective in everyday populations over time as a result of inappropriate prescriptions, unnecessary use, or their overuse in both humans and animals. At the same time, research and development on new antibiotics has slowed down [2]. It is to note that almost all the new antibiotics that have been brought to market in recent decades are variations of antibiotic drugs classes that had been discovered by the 1980s.

The European Surveillance of Antimicrobial Consumption Network showed that large variations in antibiotic use and antibiotic resistance rates exist across Europe, with higher use in Southern Europe including France and lower in Northern Europe [3].

In general, social media refers to forms of electronic communication (such as websites for social networking and microblogging) through which users create online communities to share information, opinions, personal messages, photos, videos, and other contents within internet apps [4]. Laudon and Traver [5] describe online social networking as an online social area for people who share common ties that can interact with one another. Organizations are able to use the internet to connect with consumers in the health care field. Consumers heavily rely on information found online and use internet to gather health care information and connect with other patients to garner support and learn about similar conditions. Individuals will use social media to post reviews or other comments that support or possibly deter others from choosing that type of health care in the future. It is essential for providers to be active on social media and provide accurate information and connect with readers. Certain groups use online channels more often depending on the topic they are searching for. And people responsible for others, such as parents or caregivers, search health topics even more. Almost 90% of adult users accessed popular social media sites to find and share health information [6]. People aged 18 to 24 years are twice as likely to use social media to discuss health issues than people aged between 45 to 54 years [7].

The digitizing of health care data, as well as advancements in computer processing and data storage, has enabled the development of advanced algorithms in the form of artificial intelligence (AI) [8]. AI tools are strongly related with data mining and AI is nowadays ranked among the top-10 technology, whichever the application [9]. Despite their limitations, AI tools and techniques that are still in their infancy already provide substantial benefits in providing in-depth knowledge on individuals’ health and predicting population health risks. Their use for medicine and public health is expected to increase substantially in the near future [10].

Health is a multifaceted concept and there is no single indicator that can adequately assess its impact in relation to quality of life (QoL). The framework for measuring the QoL includes, among others, information on the share of the population with unmet needs for medical and dental care and a contrasting set of health determinant indicators [11]. In this study, we collected on the web and sought to analyze the messages and complaints of patients with antibiotic treatment failure using innovative tools, without prejudging the cause of the failure (misuse or antibiotic resistance) in order to identify the main topics of discussion, and to identify the difficulties encountered by patients regarding antibiotic ineffectiveness and the impact on their quality of life (physical, psychological, social, or financial).

## Methods

### Study Design and Population

**Figure 1.**
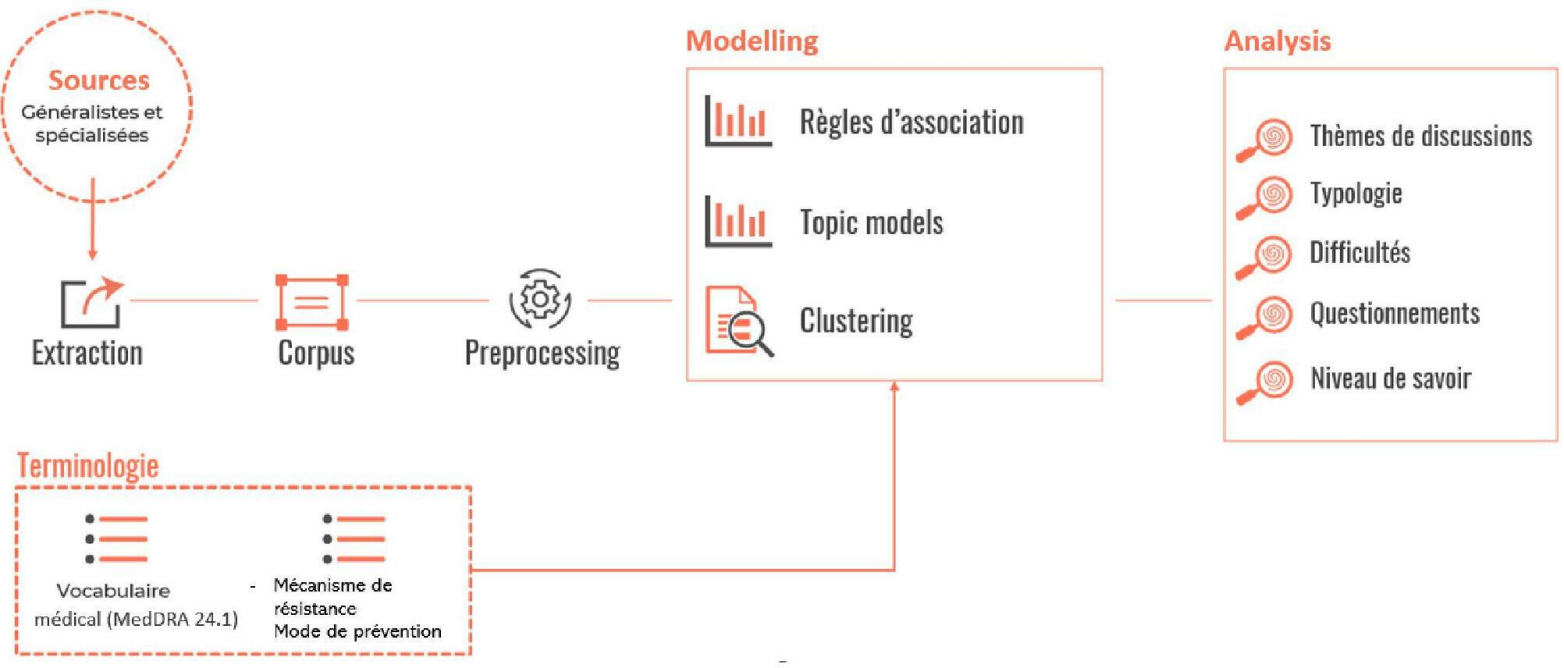
Study framework.

This was a noninterventional retrospective study using a text mining approach to retrieve information from social media posts (data available in the public domain) written by French speaking internet users between 2014 and 2020 (Figure 1). The study was conducted in 2 phases, data collection using the published Detec’t webcrawler [12,13] developed by Kap Code (Paris, France) to collect antibiotic resistance-related posts and quantitative and qualitative analyses to identify trends and characterize key topics discussed by French speaking users.

### Data Extraction

A web crawler is an engine that browses through hyperlinks and stores them for future download of associated web pages (identified by the visited hyperlinks) [14]. Scraping of messages was performed according to the HTML structure of each forum. All discussions containing at least one of the keywords or one of their synonyms, coupled with the search for a lexical field of inefficiency within the same message (eg, inefficient or does not work), were automatically retrieved with all the associated metadata and anonymized before being stored in a study-specific database.

Messages associated with antibiotic’s supposed ineffectiveness were retrieved from 46 French sources (such as Doctissimo [Reworld Media], Twitter [rebranded as X], and listed in Table 1) dating from 2014 to 2020.

**Table 1.** Data sources. Messages about lack of efficacy of antibiotics and antibiotic resistance were retrieved from 46 French sources.

Forums	Posts, n	Users, n
Twitter (X)	888	475
Doctissimo	885	621
Atoute	396	131
Futura-Sciences	260	135
Au féminin	171	126
Meamedica	125	124
Les impatientes	123	54
Psychologies	94	43
Onmeda	94	85
Journal des femmes	79	62
Maladies rares	77	67
Thyroide	72	46
YouTube	71	68
Psychoactif	68	42
Lymphome espoiri	61	41
Vinted	53	40
Vulgaris Medical	49	38
Ligue contre le cancer	47	43
Andlil	39	18
Alarm.asso	38	15
Maman pour la vie	37	29
Pedia Blog	13	7
Forum HardWare	10	7
Other sites	23	18
Total	3773	2335

**Figure 2.**
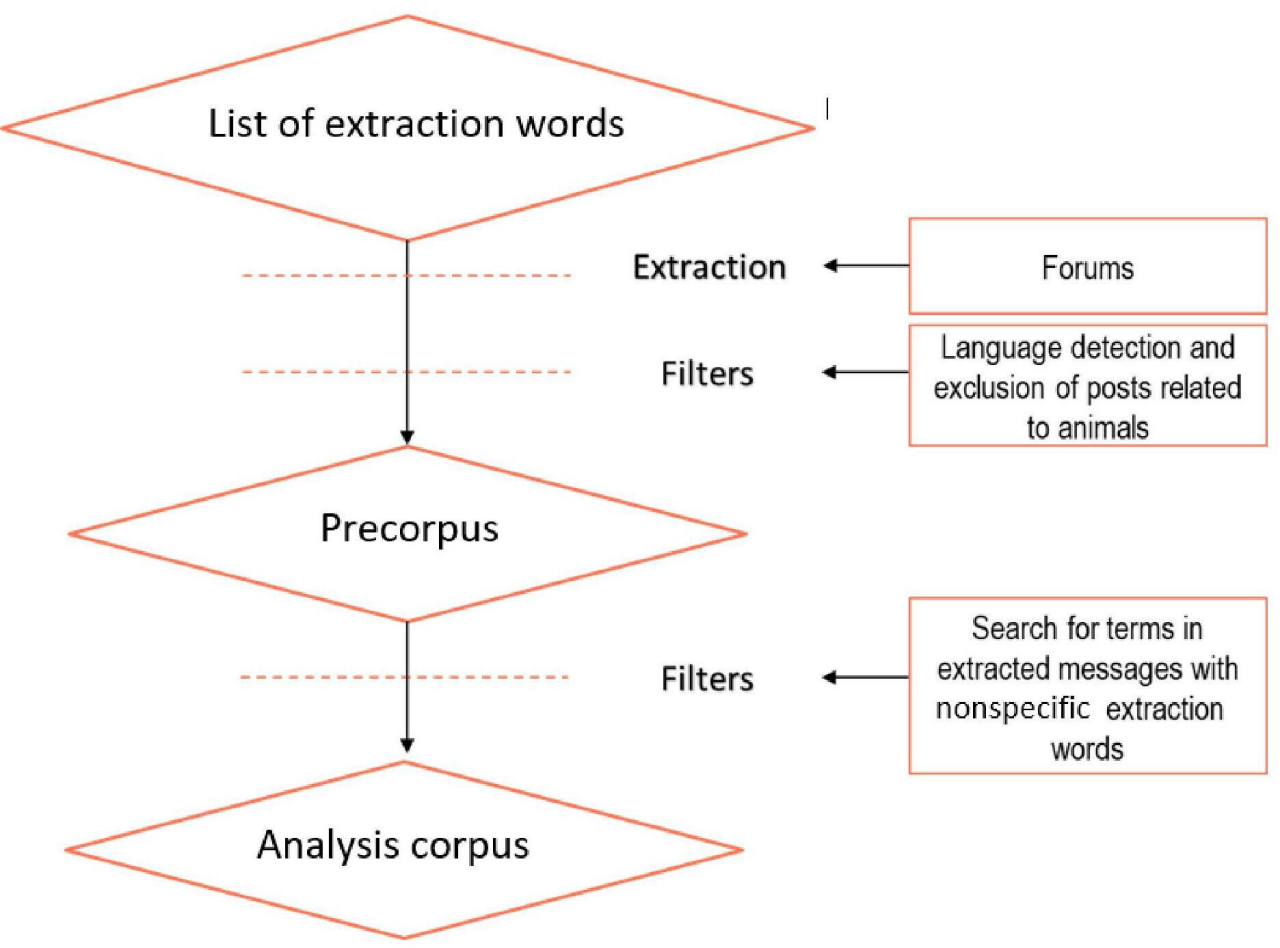
Flowchart presenting the steps for creating the analysis corpus.

The analysis corpus (Figure 2) consisted of the corpus filtered after the removal of messages containing predetermined keywords written in a language other than French, post containing animal-related vocabulary, and messages containing at least 1 of the study-specific exclusion words listed in Multimedia Appendix 1.

### Data Analysis

#### Age and Gender

Web users’ gender is determined through the identification of regular expressions for each gender. First names and gender-associated suffix and prefix are first searched in the username. Then, content of all available messages is screened for gender-specific lexical fields and gender agreements of adjectives and verbs. Finally, a score is computed for each gender and a prediction for the user is obtained by comparing them.

Web users’ ages are identified by a 2-fold method. First, regular expressions of age are identified in the total of the user posts. Then, if no expressions were found, a probabilistic machine learning model predicts an age based on several features. Among them, the model considers syntactic aspects of posts as well as expressed feeling and the source on which the user expresses himself.

#### Topic Model

A topic model was applied to identify the themes addressed in each message. Topic models consist of text mining approach aiming to automatically identify the abstract themes addressed in a collection of documents. Such models are based on the hypothesis that each document in the corpus corresponds to a distribution of several topics. A biterm topic model (BTM) was used to identify the topics without previous knowledge. A topic is defined as a subject of discussion, which amounts to tokens that frequently appear together in a corpus. The BTM considers the whole corpus as a mixture of topics, where each posts’ co-occurring pair of tokens (the biterm) is drawn from a specific topic independently, and modeled topics are probability distributions over the biterms [15].

Topics being probability distributions over tokens of the corpus of study, they can be characterized by the highest per-topic probability tokens. Weighting these probabilities through term-frequency inverse document frequency weighting allows to allocate a higher importance to topic-specific tokens. In this case, the per-topic probability of a token was weighted by the inverse of the probabilities of this token in other topics. For each topic, tokens were ranked from highest to lowest weighted probabilities. The first 15 tokens are designated as the set of characteristic tokens and used to manually name the topic.

#### QoL

QoL impacts were identified; thanks to an algorithm previously developed [16] on the basis of 2 standardized questionnaires measuring health-related QoL (EQ5D and SF36). The algorithm is 2-fold. First, it indicates if an impact is expressed and, second, thanks to 5 specific models, it indicates the nature of the impact (physical, psychic, activity-related, relational, or financial). The 6 models (1 for the first step and 5 for the second) are extreme gradient boosting models trained on annotated patients posts related various pathologies. Features involved in both steps of the algorithm describe expressed feelings, grammar, conjugation, and lexical fields of health-related QoL-related features, integrating semantic fields specific to the social media domain, allowing it to capture the patient experience as closely as possible. This algorithm was used to automatically create groups of posts as per type of impact, which were subsequently manually reviewed.

### Ethical Considerations

This study included data from publicly available sources; private groups and web pages were thus excluded from our data extraction process. We did not seek approval as users automatically grant their consent for the reuse of their data when they post on public platforms. Furthermore, the results of this study do not contain any identifiable information and are presented in aggregate. Information such as the name, username or handle, geographic locations, or any other sensitive data were not included.

## Results

### Description of the Population and Posts

**Figure 3.**
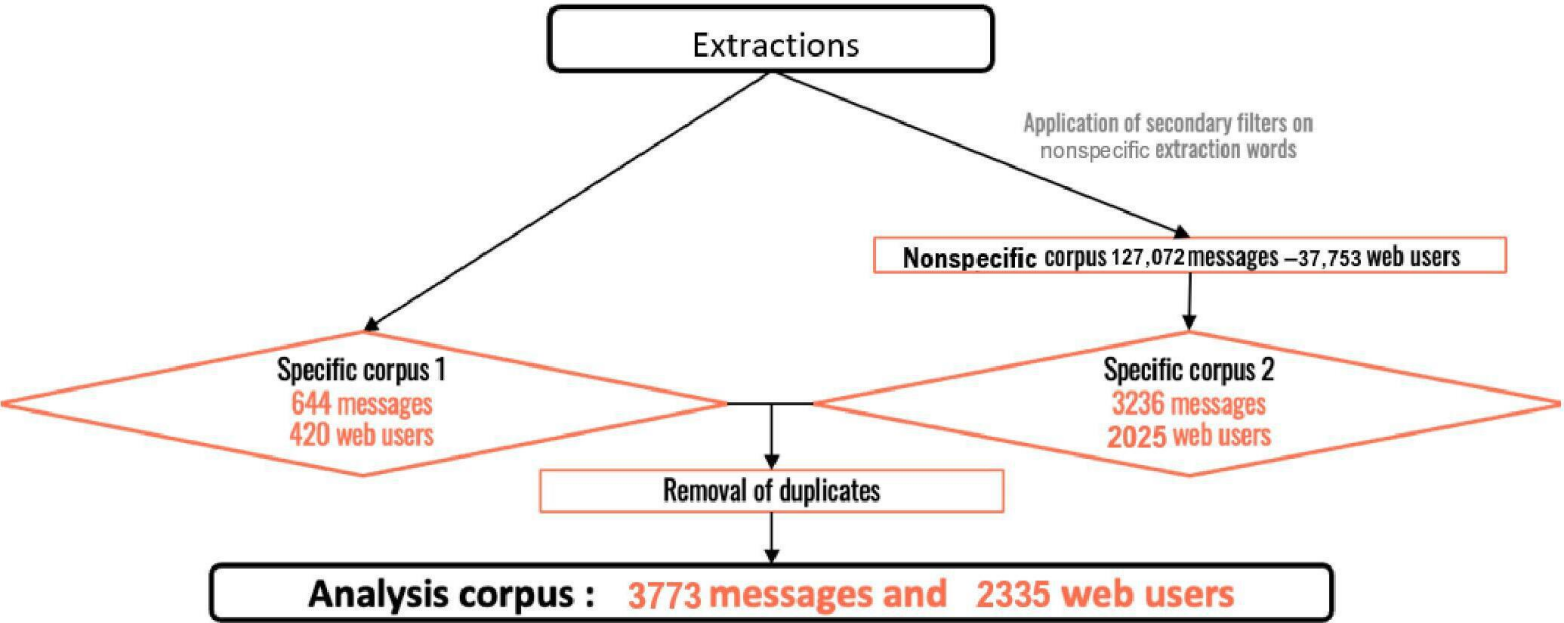
Methodology.

After filtering and formatting, the analysis corpus contained a total of 3773 messages corresponding to 2335 different web users, with a mean of 1.62 posts per user (Figure 3).

Data showed that there is no specific forum for the sharing of information on activities relating to lack of efficacy of antibiotics and antibiotic resistance and a disparity of sources with a significant proportion of extracted data coming from X and Doctissimo with 888 (23.5%) and 885 (23.5%) posts out of 3773, respectively (Table 1). Furthermore, there was no connection between sources, suggesting a lack of structuring and of visibility of information networks.

**Figure 4.**
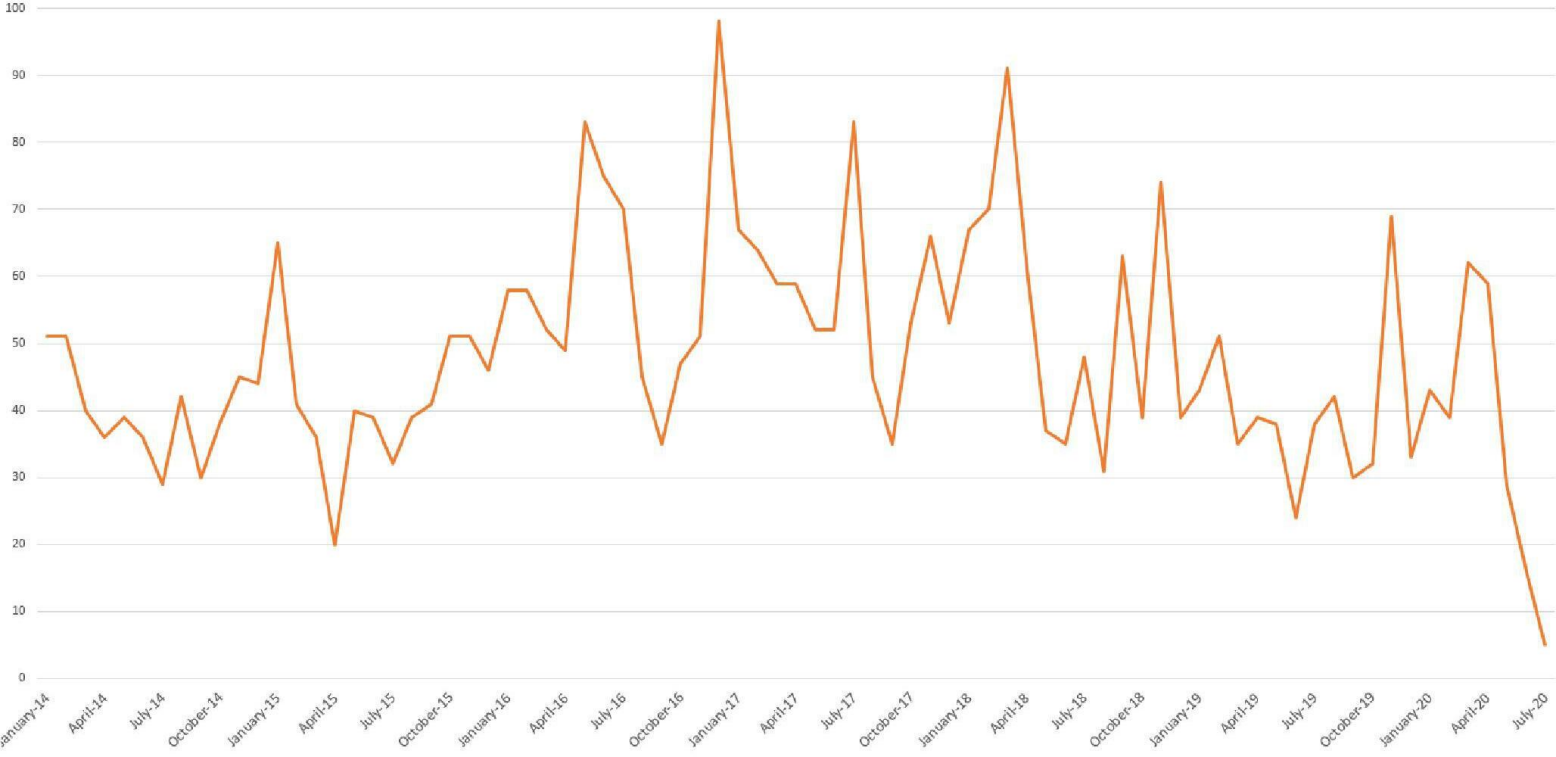
Fluctuations in the evolution of the volume of messages between January 2014 and July 2020 .

We observed fluctuations in the evolution of the volume of messages between January 2014 and July 2020 (Figure 4). We observe a seasonality effect mainly during the “cold” and flu periods, from November to March.

**Figure 5.**
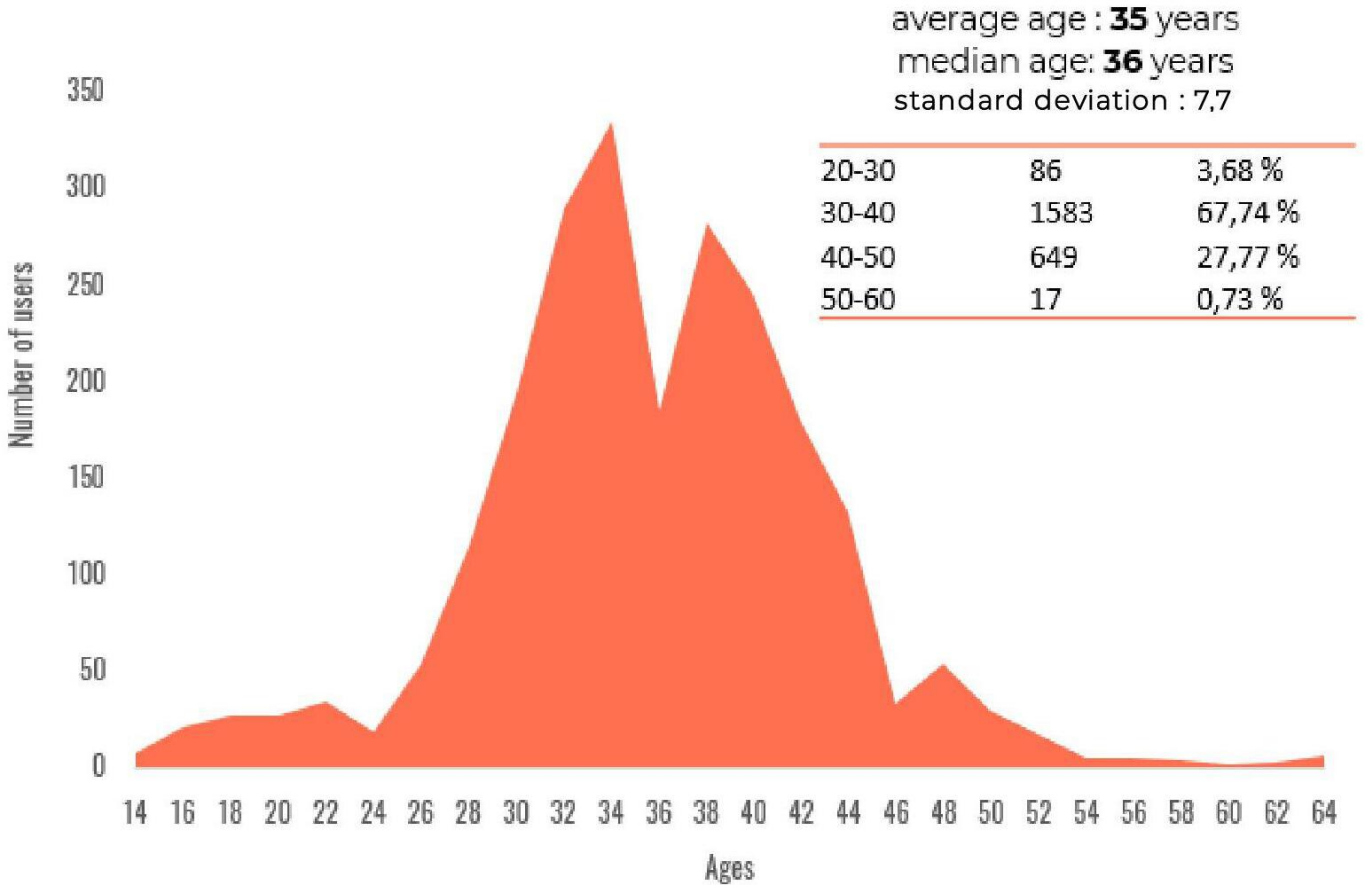
Age distribution.

Most users were determined as being women (2015/2337, 86.22%), and the average age was estimated of about 35 years (Figure 5). This is consistent with the results of many studies that point out that women express more personal issues in social networks [17,18]. Furthermore, mothers are more likely to seek advice and help about their infants’ health from multiple sources, including social media [19].

#### Discussion Themes and Topics

As shown in Table 2, the BTM identified 13 main themes of discussion. The largest category of topics was related to the inefficacy of treatment options (“ineffective antibiotics” [597/3770, 15.84%]), especially for ambulatory diseases such as cystitis (356/3770, 9.44%), acne (226/3770, 5.99%), or dental disorders (109/3770, 2.89%), followed closely by the lack of information regarding their use (“antibiotics and pregnancy,” 402/3770, 10.66%). Furthermore, our findings showed a certain degree of superficiality in the knowledge regarding antibiotics resistance (351/3770, 9.31%) and antibiotics overuse (301/3770, 7.98%).

**Table 2.** Distribution of posts by overall topic category. Application of a biterm topic model that automatically identifies the different topics addressed within the message corpus. Each theme is associated with a set of characteristic words which allows to define them. A message can only be associated with 1 theme.

Topics	n (%)
Ineffective antibiotics	597 (15.84)
Antibiotics and ear, nose, and throat disorder	461 (12.23)
Antibiotics and pregnancy	402 (10.66)
Persistent urinary infections	356 (9.44)
Mechanisms of resistance	351 (9.31)
Antibiotics overuse	301 (7.98)
Inappropriate use of antibiotics	272 (7.21)
Medical data exchanges	238 (6.31)
Antibiotics and acne	226 (5.99)
Gastrointestinal impact of antibiotics	137 (3.63)
Dental disorders and antibiotics	109 (2.89)
Sensitivity analysis antibiogram	77 (2.04)
Nosocomial infection	66 (1.75)

#### QoL

QoL is an important criterion for evaluating the effects of a disease and of treatment interventions. In this study, the personal experience of the users in terms of impact on QoL was assessed as physical well-being, social well-being, financial well-being, emotional or psychological well-being, and day-to-day activities. Among the 3773 messages posted, 63% (2384) contained at least 1 impact related to the ineffectiveness of antibiotic treatment on their daily life (Table 3). The most reported impact is physical (1866, 78% of messages), with patients discussing the persistence of symptoms (pain and fatigue), and adverse effects (diarrhea and joint pain). Psychological impact (1551, 65% of messages) is characterized by feelings of anxiety, fear, or despair, with some messages even mentioning depression. Current activities (745, 31% of messages) were also impacted, with sick leave, professional difficulties, or a decrease in regular physical activities. Social impact (602, 25% of messages) was marked by family or sexual problems and feelings of social isolation. Financial impact was less discussed in only 1% (28) of the messages, with messages essentially related to unnecessary expenses or care coverage.

**Table 3.** Impact of antibiotic treatment ineffectiveness on patients’ quality of life.

Impact	n (%)
Physical	1866 (39)
Psychological	1551 (32)
Day-to-day activities	745 (16)
Social	602 (13)
Financial	28 (1)

## Discussion

### Principal Findings

Using a web-based tool, we have been able to assess retrospectively how (web user) patients perceive lack of efficacy of antibiotics in France over the last 7 years. Most users were women, and the average age was of about 35 years (Figure 5). Discussions were mainly about therapeutic wandering, without a strong knowledge about antibiotics mechanism of action and antibiotic resistance. This situation might be due to some lack of knowledge and interest on the subject, highlighting the wandering without addressing the essential points at the origin of the lack of efficacy like prescription of antibiotics in case of viral infection. Furthermore, we showed that there is a strong impact of perceived ineffectiveness of antibiotic therapy on the QoL of the patients, especially a strong physical and psychological impact for diverse infections and situations.

### Limitations of the Study

This study is to our knowledge the first analysis of messages posted on social media and related to antibiotic resistance in human medicine. It also shows several limitations. First, by focusing only on terms in the French language, we will have underreported the total volume of messages related to antibiotics resistance, and thus, these results are not generalizable at a worldwide scale. Furthermore, using social media to analyze patients’ reactions excludes patients who do not have access to the internet or who are not familiar with the use of online discussions.

Extraction bias and a limited amount of data compared with similar studies related to other patient groups (such as breast cancer) were the second limitation. Even if a reasonable sized set of keywords was used, keyword selection in social media studies can induce varying levels of extraction bias, accentuated by a low volume of data.

Another limitation of using social media is the lack of complete information about individual cases. There is also the problem of discovering demographic information—only limited or no information regarding individual user demographics (such as age and origin) may be available.

Despite these limitations, social media represent an ideal place where patients can freely and spontaneously discuss their experiences along with their therapy, thus providing valuable information on their QoL difficult to access by other means. Nevertheless, these observations should be interpreted cautiously, since social media data may include a higher frequency of erroneous information, and patients posting on social media forums may not be representative of the wider patient population.

### Implications and Future Research

Messages published on social networks should be integrated into the assessment of patient’s QoL, as they can help to characterise the patient’s experience in a more individualized and spontaneous way. Furthermore, it seems important to further explore certain subpopulations (eg, pregnant women, children, or even older adults via their parents or caregivers).

To the extent that the most dominant topics can be interpreted as unmet informational needs, our study highlights the refinement of practical implications such as the improvement of existing tools, and further reinforcement of the use of available tools (such as antibiotic susceptibility testing or rapid detection tests), and as well as the communication around them.

The ability of internet users to attribute this perceived ineffectiveness to inadequate prescribing or antibiotic resistance is not perceptible in this study and could be the subject of further analysis.

This study suggested to carry out a study segmented by year to examine the evolution of the different subjects (QoL, topics, and treatments) over the years and to observe the global evolutions. Furthermore, similar studies in another country, such as Germany or the United Kingdom, should be planned to compare messages posted between three countries with similar way of life and growth domestic product.

### Conclusion

Given that health information is shared extensively on social networks, such services can potentially be used to gather important real-time health data and may provide a venue to identify potential misuse or misunderstanding of antibiotics, promote positive behavior change, and disseminate valid information related to QoL and to bacterial and nonbacterial infections.

One of the most important challenge for health care professionals is to educate people about antibiotics, their adverse effects, and to encourage them to stop the misuse of such drugs. Our study illustrates the current situation concerning the lack of efficacy of antibiotics and the paucity of valuable information and treatment options for wandering patients. In our case, nothing allows us to affirm that these are therapeutic failures related to antibiotic resistance, inappropriate prescriptions or misuse by patients. However, it can provide health care experts with direct patient input on their experience in real-life conditions. This web analysis made it possible to collect, for the first time, the expression of patients who have failed antibiotic therapy. While the extent of these failures is difficult to demonstrate, these patients’ experiences tell us how they feel. This research deserves to be continued to better understand the evolution of this phenomenon.

## Supplementary material

10.2196/37160 Multimedia Appendix 1 List of exclusion words used to filter the extraction of messages.

## Abbreviations

AI: artificial intelligence
BTM: biterm topic model
QoL: quality of life

## References

[1] Sengupta S, Chattopadhyay MK, Grossart HP (2013). The multifaceted roles of antibiotics and antibiotic resistance in nature. Front Microbiol.

[2] Ventola CL (2015). The antibiotic resistance crisis: part 1: causes and threats. P T.

[3] European Centre for Disease Prevention and Control (2022). Antimicrobial resistance in the EU/EEA (EARS-net) - annual epidemiological report for 2020.

[4] von Muhlen M, Ohno-Machado L (2012). Reviewing social media use by clinicians. J Am Med Inform Assoc.

[5] Laudon KC, Traver CG (2008). E-Commerce: Business, Technology, Society.

[6] Tennant B, Stellefson M, Dodd V (2015). eHealth literacy and Web 2.0 health information seeking behaviors among baby boomers and older adults. J Med Internet Res.

[7] Harger howe advertising. Top healthcare recruitment social media secrets.

[8] AHRQ (2011). Medal.org (Medical Algorithms): Agency for Healthcare Research and Quality, Innovation Exchange.

[9] (2020). 10 Tech & IT Buzzwords For 2021 You Won’t Be Able To Avoid BI Blog | Data Visualization & Analytics Blog | datapine.

[10] Shaban-Nejad A, Michalowski M, Buckeridge DL (2018). Health intelligence: how artificial intelligence transforms population and personalized health. NPJ Digit Med.

[11] https://ec.europa.eu/eurostat/statistics-explained/index.php?title=Archive:Quality_of_life_indicators_-_health..

[12] Abdellaoui R, Schück S, Texier N, Burgun A (2017). Filtering entities to optimize identification of adverse drug reaction from social media: how can the number of words between entities in the messages help?. JMIR Public Health Surveill.

[13] Kürzinger ML, Schück S, Texier N (2018). Web-based signal detection using medical forums data in France: comparative analysis. J Med Internet Res.

[14] Najork M, LIU L, ÖZSU MT (2009). Encyclopedia of Database Systems.

[15] Yan X, Guo J, Lan Y, Cheng X A biterm topic model for short texts. https://dl.acm.org/doi/proceedings/10.1145/2488388.

[16] Renner S, Marty T, Khadhar M (2022). A new method to extract health-related quality of life data from social media testimonies: algorithm development and validation. J Med Internet Res.

[17] Rollero C, Daniele A, Tartaglia S (2019). Do men post and women view? The role of gender, personality and emotions in online social activity. Cyberpsychology (Brno).

[18] Social Media Today Gender-Specific Behaviors on Social Media and What They Mean for Online Communications.

[19] Duggan M, Lenhart A, Lampe C, Ellison N (2015). Parents and social media. Pew Research Center.

