# Supplementary material for: Discussions of Antibiotic Resistance on Social Media Platforms: Text Mining and Mixed Methods Content Analysis Study
Source: JMIR Form Res. 2025 Apr 25;9:e37160. doi: 10.2196/37160 (PMC12047853; doi:10.2196/37160)
Supplement: Multimedia Appendix 1 [file formative-v9-e37160-s001.docx]

Appendix 1 - List of exclusion words used to filter the extraction of messages.

| \| ADAPTE \| \| --- \| \| ANTIBIO ADAPTE \| \| ANTIBIO EFFICACE \| \| ANTIBIO EST EFFICACE \| \| ANTIBIO FONT EFFET \| \| ANTIBIO MARCHE \| \| ANTIBIO MARCHENT \| \| ANTIBIO SONT EFFICACE \| \| ANTIBIOS ADAPTE \| \| ANTIBIOS EFFICACE \| \| ANTIBIOS EST EFFICACE \| \| ANTIBIOS FONT EFFET \| \| ANTIBIOS MARCHE \| \| ANTIBIOS MARCHENT \| \| ANTIBIOS SONT EFFICACE \| \| ANTIBIOTIQUE ADAPTE \| \| ANTIBIOTIQUE EFFICACE \| \| ANTIBIOTIQUE EST EFFICACE \| \| ANTIBIOTIQUE FONT EFFET \| \| ANTIBIOTIQUE MARCHE \| \| ANTIBIOTIQUES EFFICACE \| \| ANTIBIOTIQUES FONT EFFET \| \| ANTIBIOTIQUES MARCHENT \| \| ANTIBIOTIQUES SONT EFFICACE \| \| EFFICACITE ANTIBIO \| \| EFFICACITE DES ANTIBIO \| \| EFFICACITE DES ANTIBIOTIQUES \| \| SONT UTILE \| \| SUIS GUERI \| |  |
| --- | --- | --- | --- | --- | --- | --- | --- | --- | --- | --- | --- | --- | --- | --- | --- | --- | --- | --- | --- | --- | --- | --- | --- | --- | --- | --- | --- | --- | --- | --- |
|  |  |
|  |  |
|  |  |
|  |  |
|  |  |
|  |  |
|  |  |
|  |  |
|  |  |
|  |  |
|  |  |
|  |  |
|  |  |
|  |  |
|  |  |
|  |  |
|  |  |
|  |  |
|  |  |
|  |  |
|  |  |
|  |  |
|  |  |
|  |  |
|  |  |
|  |  |
|  |  |
|  |  |
|  |  |
|  |  |
|  |  |
|  |  |
|  |  |
|  |  |
|  |  |
|  |  |
|  |  |
|  |  |
|  |  |
|  |  |
|  |  |
|  |  |
|  |  |
|  |  |
